# Short-term forecasting of *M*_max_ during hydraulic fracturing

**DOI:** 10.1038/s41598-022-15365-6

**Published:** 2022-07-22

**Authors:** Ziyan Li, David Eaton, Jörn Davidsen

**Affiliations:** 1 Department of Geoscience, University of Calgary, Calgary, AB T2N 1N4 Canada; 2 Department of Physics and Astronomy, University of Calgary, Calgary, AB T2N 1N4 Canada; 3 Hotchkiss Brain Institute, University of Calgary, Calgary, AB T2N 4N1 Canada

**Keywords:** Geophysics, Seismology

## Abstract

Previous studies of injection-induced earthquake sequences have shown that the maximum magnitude (*M*_*max*_) of injection-induced seismicity increases with the net injected volume (*V*); however, different proposed seismic-hazard paradigms predict significantly different values of *M*_*max*_. Using injection and seismicity data from two project areas in northeastern British Columbia, Canada, where hydraulic fracturing induced seismicity was observed, we test the predictive power and robustness of three existing and one novel method to estimate *M*_*max*_. Due to their vastly different values of seismogenic index (Σ), these two project areas represent end-member cases of seismogenic response. Our novel method progressively adjusts the *M*_*max*_ forecast under the assumption that each recorded event embodies an incremental release of fluid-induced stress. The results indicate that our method typically provides the lowest upper bound of the tested methods and it is less sensitive to site-specific calibration parameters such as Σ. This makes the novel method appealing for operational earthquake forecasting schemes as a real-time mitigation strategy to manage the risks of induced seismicity.

## Introduction

Earthquakes can be induced by anthropogenic activities, such as enhanced geothermal systems^1–3^, hydraulic fracturing^4,5^, and saltwater disposal^6,7^. In North America, saltwater disposal has been the dominant cause in some regions such as the central U.S.^8,9^, whereas hydraulic fracturing (HF) is one of the major causes of induced seismicity in western Canada^10,11^. HF operations typically produce microearthquakes^4,12^ with moment magnitude (*M*_*W*_*)* < 0 and thus have been considered to play a relatively minor role in regional seismicity rate^13^. However, events up to *M*_*W*_ ~ 4.6 have been induced by HF in western Canada, including the Cardston swarm^10^, the Fox Creek area^10^, and Fort St. John earthquake sequence^14^. Proposed models for fault activation by HF include pore-pressure increase, poroelastic stress changes or aseismic fault slip^15^, and pre-existing fracture systems may play an important role in connecting HF operations to critically stressed faults^16,17^^.^

Estimation of the upper-bound magnitude (*M*_*max*_) expected for a given HF operation is an essential element of hazard assessment for induced seismicity^18^. Further, short-term forecasting of *M*_*max*_ has the potential to inform real-time mitigation strategies that are required in some monitoring systems for induced seismicity^19^. In cases of runaway rupture, wherein the earthquake rupture zone expands beyond the bounds of the stimulated region^1^, *M*_*max*_ is ultimately limited by tectonic parameters such as stress state and the total fault area^20^. These parameters may be difficult to determine in areas with low natural seismicity rates. In the more common arrest rupture scenario, where the seismic rupture zone lies inside the stimulated region of a fault^21^, various paradigms and models have been proposed that link *M*_*max*_ to the net injected fluid volume^22^. In this paper, we aim to establish a robust method that can be applied for real-time, short-term forecasting. We focus on approaches to estimate an upper bound for the expected magnitudes, as in other previously published papers. Specifically, the goal of this paper is threefold: (1) Introduce a modified method that takes additional physical insight into account to provide an improved estimate of *M*_*max*_. (2) Compare the performance of different proposed methods across case studies that can be considered end members in terms of seismogenic response. (3) Test the parameter sensitivity of different proposed methods to establish their usability for real-time feedback for fluid injection operations.

Our present study considers the arrested-rupture scenario and compares estimates obtained using three previous models for *M*_*max*_*.* As elaborated in the next section, method (1)^23,24^, herein referred to as an uncalibrated moment cap formulation, posits that seismic moment has an upper bound equal to the product of net injected fluid volume with fault rigidity (shear modulus). In practice, observed maximum seismic moments are typically orders of magnitude smaller than this uncalibrated estimate, implying that most of the induced deformation is aseismic^25,26^ (i.e., too slow to radiate elastic waves in the frequency band of conventional seismic measurements). Accordingly, method (2) is a modified version of the first method and applies an additional calibration factor called the seismic efficiency ratio (S_EFF_)^27^. Finally, using the concept of the seismogenic index (∑) to associate the total injected volume with the number of induced events, method (3) provides an upper bound for *M*_*max*_ based on the observed frequency-magnitude relation^20^. We remark that existing methods are usually sensitive to calibration parameters, such as seismogenic index, *S*_*EFF*_, and *b* value. Further, these methods yield monotonically increasing *M*_*max*_ with respect to accumulated volume (or time), often resulting in the overestimation of *M*_*max*_. The upper limit of induced seismicity can also be estimated by a fracture-mechanics-based model^21^, however, it requires several parameters that are generally not well constrained in practice. Further, for certain parameter choices, the forecasted *M*_*max*_ by this method is very similar to method (3), including the slope of the relationship between injected volume and cumulative seismic moment. Given all this, we introduce a new method to forecast *M*_*max*_ for injection-induced seismicity, and cross compare our method with method (1)–(3).

As in method (2), our approach determines an upper bound for the cumulative seismic moment, using a calibration process to estimate the *S*_*EFF*_ parameter. Unlike method (2), we obtain a time-dependent forecast for the maximum seismic moment by taking the difference between the estimated upper bound of the cumulative seismic moment and the observed cumulative seismic moment. In our proposed approach, this difference represents a time-varying seismic moment deficit; thus, each recorded event represents an incremental release of stress that competes with increasing injected volume during fluid injection^28^.

To evaluate the methods’ accuracy, we perform hindcast calculations using seismicity and HF injection data from two project areas in northeastern British Columbia (BC), Canada. In project area one, multistage HF operations were carried out in 14 closely spaced horizontal wells over a time span of four months. Concurrent active HF operations in multiple wells resulted in semi-continuous injection throughout this time. In project area two, HF operations moved between different well pads and were implemented episodically over a time span of about 21 months, with pauses of 3–11 months between each operational episode.

Comparison of the results using the four methods in both project areas shows that our novel method provides the lowest, and often most accurate, upper bound on maximum magnitude. Further, our method is arguably more robust, as the parametric analysis indicates that it is less sensitive to site-dependent calibration parameters. Thus, our method provides a practical approach to obtain real-time feedback during HF operations while also being capable, in principle, to signal the onset of runaway ruptures.

## Methods

### Data

**Figure 1:**
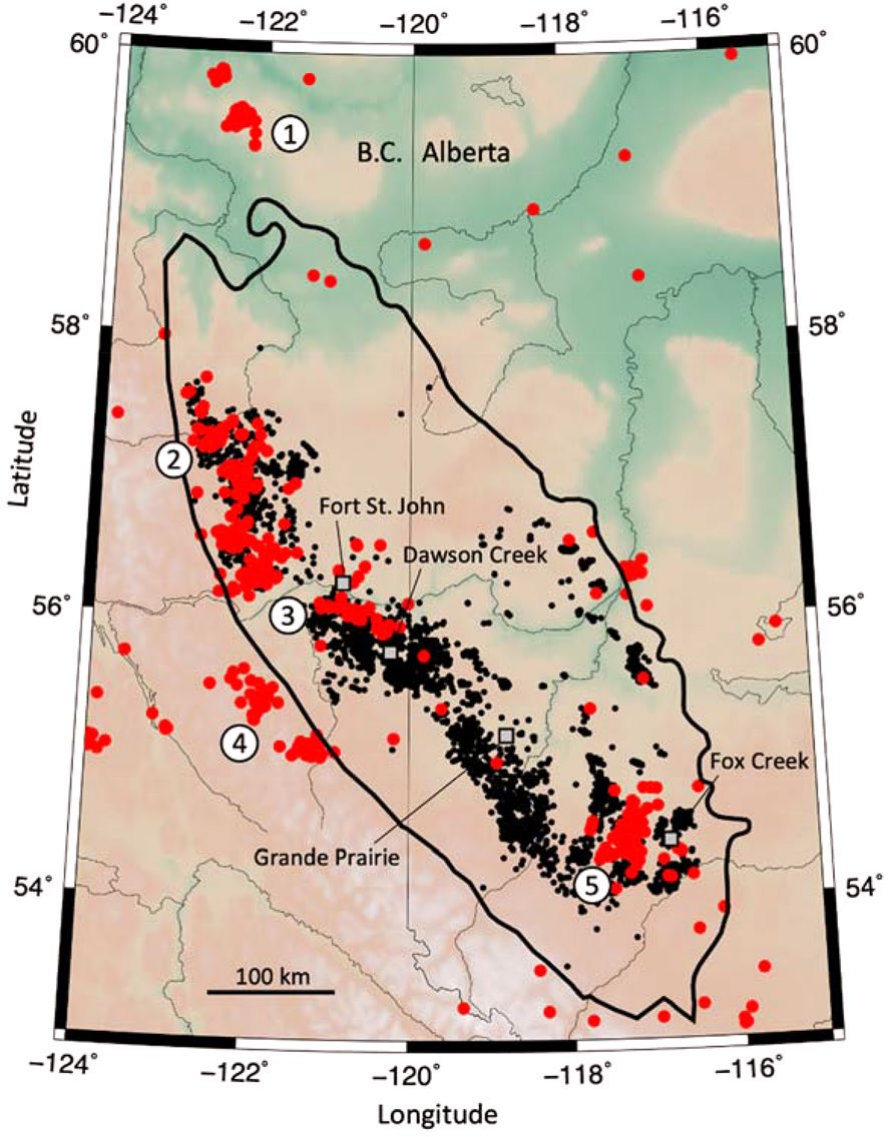
Regional map of seismicity and well distribution. Map of M_L_ ≥ 2.5 seismicity (red dots) and hydraulically fractured wells completed in the Montney formation (black dots), between October 2006 to September 2017. Towns and cities are labelled, and black outline shows the extent of the Montney play. Numbers indicate seismicity clusters: 1 = Horn River Basin (HRB), 2 = northern Montney, 3 = Kiskatinaw, 4 = quarries, 5 = Fox Creek (Duvernay). In this paper, study area 1 is located north of Fort St. John, area 2 is located to the south of Fort St. John (Modified from Schultz et al.^32^. The map was created using Generic Mapping Tools (GMT) version 6^47^).

This study focuses on two project areas near Fort St. John, British Columbia, Canada, within a region undergoing extensive unconventional resource development using multistage HF in horizontal wells^29^, as shown in Fig. 1. The target unit in both areas is the Triassic Montney Formation, a southwestward thickening wedge of planar laminated grey siltstone, up to more than 300 m thick, that has become pervasively charged with hydrocarbons^30^. Since the Montney play in BC has a relatively high geological susceptibility to induced seismicity^31,32^, oil and gas operators have deployed dense seismograph networks to detect and characterize induced microearthquakes during operations. In project area one, seismicity was monitored using an array consisting of 7 private seismograph (broadband) stations and additional 2–3 public stations with an average inter-station spacing of approximately 12 km. The sensors were buried to the depth of 1 m and consisted of either broadband three-component (3C) seismometers or 4.5 Hz three-component geophones. During commercial processing of the passive seismic data, preliminary event detection was carried out using the short-time-average/long-time-average method^33^ followed by manual inspection to search for missed events, such as due to the occurrence of events that are closely spaced in time. To create the seismicity catalog, preliminary hypocentres were estimated using a 1-D layered model, and final hypocentres were obtained using a 3D velocity model derived from joint inversion. Similar acquisition and processing methodologies were used to generate the seismicity catalogs for project area two.

In project area one, HF took place in 13 horizontal wells with 2–3 concurrent HF operations at any given time. Each well was stimulated using between 27 and 40 HF stages (a stimulated segment of the well that is isolated from the rest of the wellbore), with an average stage duration of 3.9 h and an average injected volume per stage of 2.2 × 10^3^ m^3^, leading to a total injected volume for the entire project of 1.2 × 10^6^ m^3^. A total of 2972 events were recorded, with a reported local magnitude (*M*_*L*_) range of − 0.9 to 2.16. To compute seismic moment, the magnitudes were converted from local magnitude (*M*_*L*_) to moment magnitude (*M*_*W*_) using the relation of Ross et al.^34^,

1 $$M_{\text{w}} = \, 0.{754}M_{{\text{L}}} + \, 0.{88}$$

which has been shown to provide a good fit to magnitude observations from the western Canada Sedimentary Basin^35^. After applying this transformation, the magnitude range becomes 0.2 to 2.51 MW. Moment magnitude was then converted to scalar seismic moment (*M*_0_) in units of N-m using the relationship of Hanks and Kanamori^36^,

2 $$M_{0} = { 1}0^{{{1}.{5}\left( {M{}_{W} + {6}.0{33}} \right)}}$$

**Figure 2:**
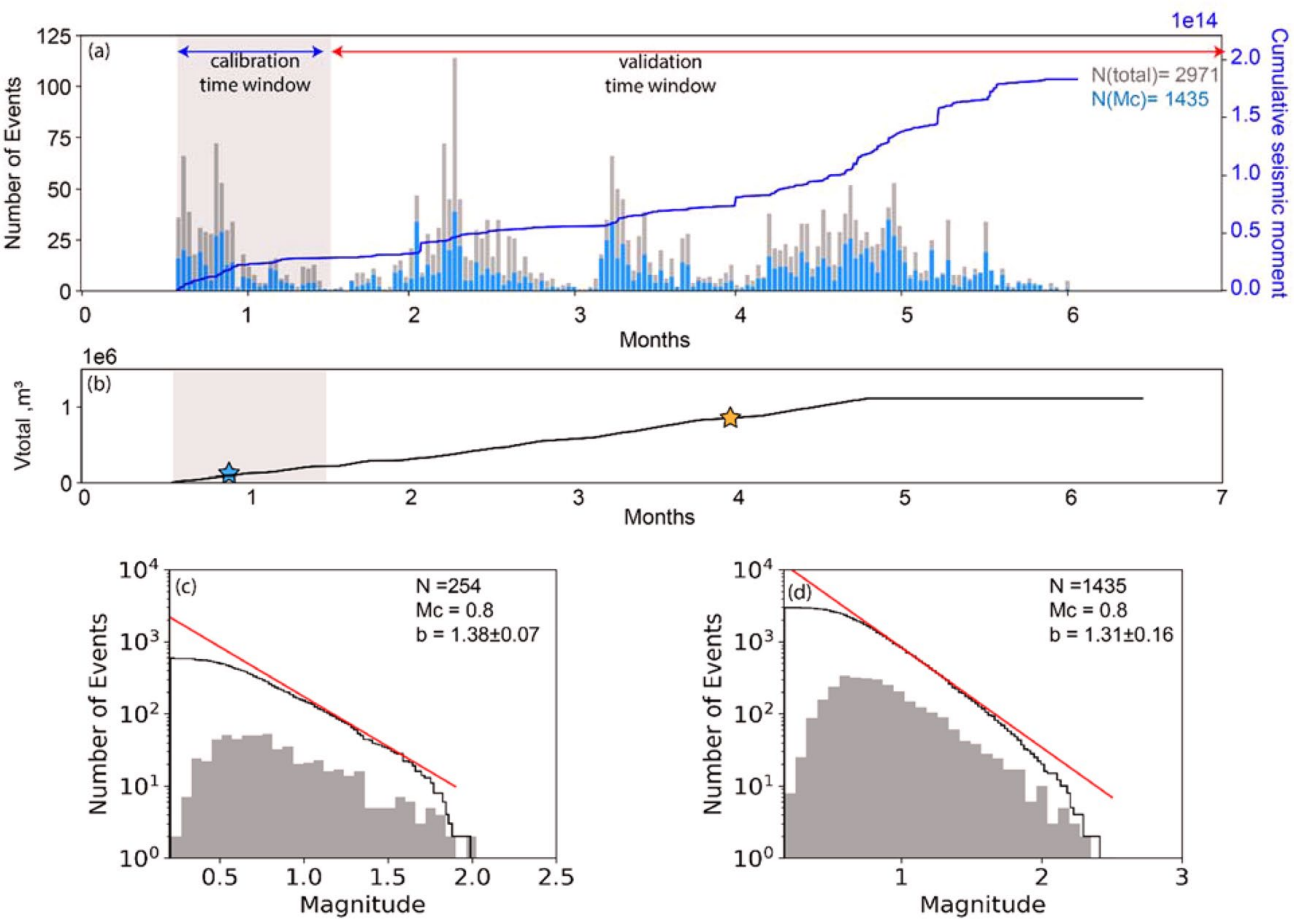
Seismic and injection history of project area one. (**a**) Histogram of the number of detected seismic events per day (grey bars for all observed seismic events and blue bars for those with magnitude larger than *M*_*c*_), and the cumulative seismic moment (solid blue line) according to injection time. (**b**) The total injection volume versus time. The blue and orange stars show the timing of the maximum moment magnitude event during injection for calibration and validation time window, respectively. The Gutenberg-Richter relation, together with *M*_*c*_ and *b* value for calibration time window and entire dataset are presented in (**c**) and (**d**), respectively. The *b* value was calculated using the maximum likelihood method^48^, where the uncertainty shows the 95% confidence limits. The magnitude of completeness (*M*_c_) was calculated using the *b*-value stability method^49^.

Figure 2a shows that, for project area one, the injection rate is nearly constant over approximately four months of the project; by contrast, the seismicity rate shows substantial variability over the course of the HF program and declined gradually for approximately 1.5 months after injection stopped. The episodic seismic moment observed during different periods is inferred to reflect varying proximity of operations to seismogenic faults. In addition, the occurrence of three relatively large events results in step-like increases in a plot of the cumulative seismic moment versus time (Fig. 2a). The Gutenberg-Richter relation, together with Mc and b value for calibration time window is presented in Fig. 2c. Using all available data (Fig. 2d), we obtained a *b*-value (slope of the semilogarithmic frequency-magnitude distribution) of 1.31; this value is higher than *b* ~ 1.0 that is typical for natural fault systems^37^, but it is less than the commonly observed values of *b* > 1.5 for operationally induced microseismicity^38^.

**Figure 3:**
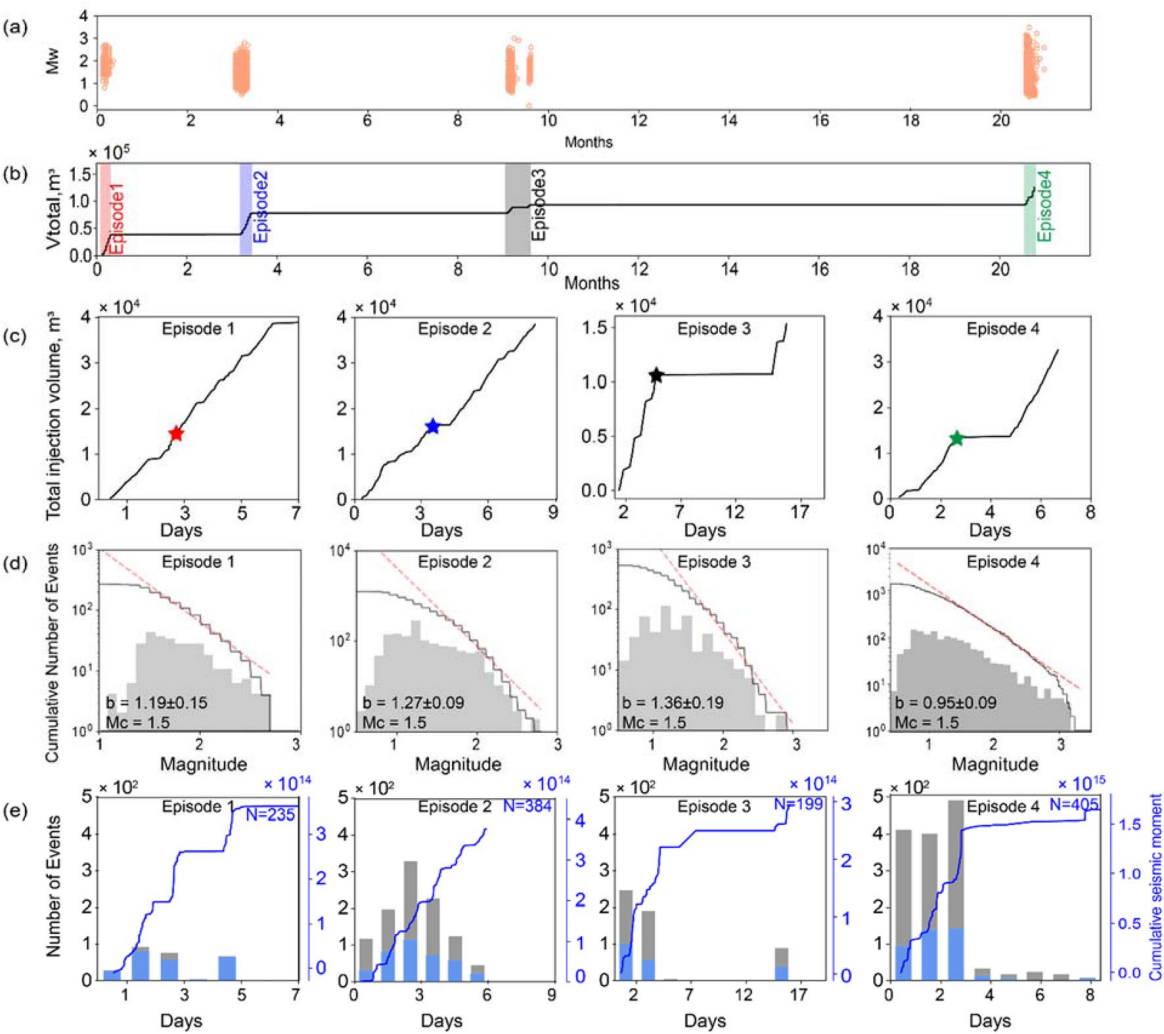
Seismic and injection history of project area two. (**a**) The moment magnitude (*M*_*W*_) of seismic events during injection (orange symbol) during the same 21-month period for the project area, based on the public catalog from the BC Oil and Gas Commission. This confirms that no significant seismicity occurred in the project 2 area between the hydraulic fracturing (HF) episodes. (**b**) Injection history in project 2 includes four injection episodes (highlighted). (**c**) The cumulative injection volume versus time for each episode. The star shows the time when the maximum magnitude event occurred during each episode. (**d**) Maximum likelihood Gutenberg-Richter relation with 95% confidence for each episode, characterized by parameters *M*_*c*_ and *b*. (**e**) Histogram of the number of total seismic events (grey) and seismic events of magnitude above Mc (blue) per day for each episode. The number of total seismic counts above Mc for each episode is labeled. The blue curve shows the cumulative seismic moment versus time for each episode.

In project area two, HF operations took place in 22 wells at a set of adjacent well pads over 21 months. The HF program was episodic and occurred within four discrete time periods, each of which lasted for 6–16 days (Fig. 3a). The number of stages (per well) ranged from 9 to 37, with injection during each stage lasting for just under one hour, on average. Summed over all four episodes, the total injected volume for project area 2 is 1.26 × 10^5^ m^3^. Due to operational differences in HF methodology employed, the average injected fluid volume per stage (~ 250 m^3^) in project area two was an order of magnitude smaller than for project area one. As noted above, high-resolution seismicity catalogs used in this study were obtained using dense seismograph arrays. The four catalogs used here cover only the time periods of active HF injection episodes, but a comparison with *M*_*L*_ > 1.5 earthquakes in the continuous seismicity catalog published by the BC Oil and Gas Commission over the entire 21-month time window of project area two shows that seismicity was indeed confined to the discrete injection intervals (Fig. 3a). In total, 3,496 were recorded, with a magnitude range of 0 to 3.48 MW after conversion of episode 4 magnitudes (originally reported as *M*_*L*_) to moment magnitude using Eq. (1). Based on the observed frequency-magnitude distributions (Fig. 3c), the *b* value for each episode ranges from 0.95 (episode 4) to 1.36 (episode 3).

### Assumptions

We consider four methods to estimate the maximum expected seismic moment, or equivalently the maximum moment magnitude (*M*_*max*_), based on the net volume of injected fluid. As previously noted, we do not consider runaway rupture scenarios (i.e., ruptures that grow beyond the stress-perturbed area of a fault^21,39^), wherein the maximum magnitude is site-specific and depends on geological factors such as fault size and stress state. We also assume that the total injected volume during HF operations within a project area, which spans multiple closely spaced horizontal wells, provides an accurate representation of the net injected fluid volume. In practice, this approach provides an upper limit for the injected volume since flowback and hydrocarbons extracted after a well goes into production are neglected. In addition, we do not consider the effects of fluid leak-off and pore-pressure dissipation over time.

### Uncalibrated moment-cap formulation [method 1]

McGarr^24^ proposed a simple formula to compute an upper bound on the cumulative seismic moment of a fluid-induced earthquake sequence, given by

3 $${\left[\sum {M}_{0}\right]}_{max}=2G\Delta V .$$

In this expression, *G* is the shear modulus, often taken to be 30 GPa, and Δ*V* is the net injected fluid volume. For a seismicity sequence characterized by a frequency-magnitude distribution with a *b*-value of 1, the largest event is expected to release 50% of the total seismic moment. For method one, combining Eqs. (2) and (3) gives an estimated upper-bound moment magnitude of

4 $${M}_{m{\text{ax}}}^{\left(1\right)}(t)=\frac{2}{3} {\text{log}}_{10}\left[G\Delta V(t)\right]-6.033 .$$

We use the superscript to *M*_max_ to specify the method number (1, 2, 3, 4). Both the net injected volume and *M*_max_ are considered to be time-dependent, meaning that future values of *M*_max_ can be forecasted based upon a planned future injection schedule.

### Calibrated moment-cap formulation [method 2]

Hallo et al.^26^ noted that slow, or aseismic, deformation likely constitutes a significant fraction of deformation processes associated with fluid injection. To account for the effects of aseismic deformation, they introduced a seismic efficiency factor (*S*_EFF_), which represents an estimate of the seismic deformation to the total deformation, leading to:

5 $${M}_{m{\text{ax}}}^{\left(2\right)}(t)=\frac{2}{3} {\text{log}}_{10}\left[{S}_{EFF} G\Delta V(t)\right]-6.033 .$$

As discussed below, *S*_EFF_ is site-specific and requires calibration; in the case of arrested rupture, it falls into the range 0 $<$ *S*_EFF_ $\le$ 1.

### Statistical formulation [method 3]

Based on sample-size statistics assuming a power-law distribution in seismic moment, Van der Elst et al.^20^ showed that the peak of the posterior probability density function for the expected maximum magnitude can be expressed as

6 $${M}_{max}^{\left(3\right)}\left(t\right)= \frac{1}{b}\left[\Sigma +{\text{log}}_{10}\Delta V\left(t\right)\right],$$

where *b* is the best-fitting semilogarithmic slope of the frequency-magnitude distribution and Σ denotes the seismogenic index, a parameter that characterizes the seismotectonic state at an injection location^40^. The seismogenic index is generally considered to be time-invariant and is defined as

7 $$\Sigma \equiv {\text{log}}_{10}N- {\text{log}}_{10}\Delta V+b{M}_{c} ,$$

where *N* is the number of seismic events with a magnitude greater than the catalog magnitude of completeness (*M*_*c*_) and Δ*V* is the corresponding injected volume. In order to apply method (3) to forecast *M*_max_ for a planned injection schedule, the parameters *b*, Σ and *M*_c_ require site-specific calibration.

### Residual calibrated moment cap formulation [method 4]

We introduce a new approach, which is a refinement of method (2). Using this method, the difference ($d{M}_{0}$) between the forecast upper limit of the cumulative seismic moment and the observed cumulative seismic moment is assumed to be stored as energy in the could that potentially be released in a single seismic event. This difference can be expressed as a residual seismic moment in the form

8 $$d{M}_{0}={S}_{EFF}2G\Delta V\left(t\right)- \sum {M}_{0}\left(t\right).$$

According to this method, at any given time the maximum magnitude for an arrested rupture is given by

9 $${M}_{max}^{\left(4\right)}\left(t\right)= \frac{2}{3}{\text{log}}_{10}\left[d{M}_{0}\right]-6.033 ,$$

where, as in method (2)^26^, *S*_EFF_ is a seismic efficiency ratio parameter that represents the ratio of seismic moment release based on the seismicity catalog to the estimated total (seismic + aseismic) moment release^26^. Once calibrated, our method progressively adjusts the forecasted maximum magnitude under the assumption that each recorded event represents an incremental release of fluid-induced stress. Consequently, a relatively large seismic event during the forecast period is generally followed by a time interval during which the operational forecast for maximum magnitude is reduced.

**Figure 4:**
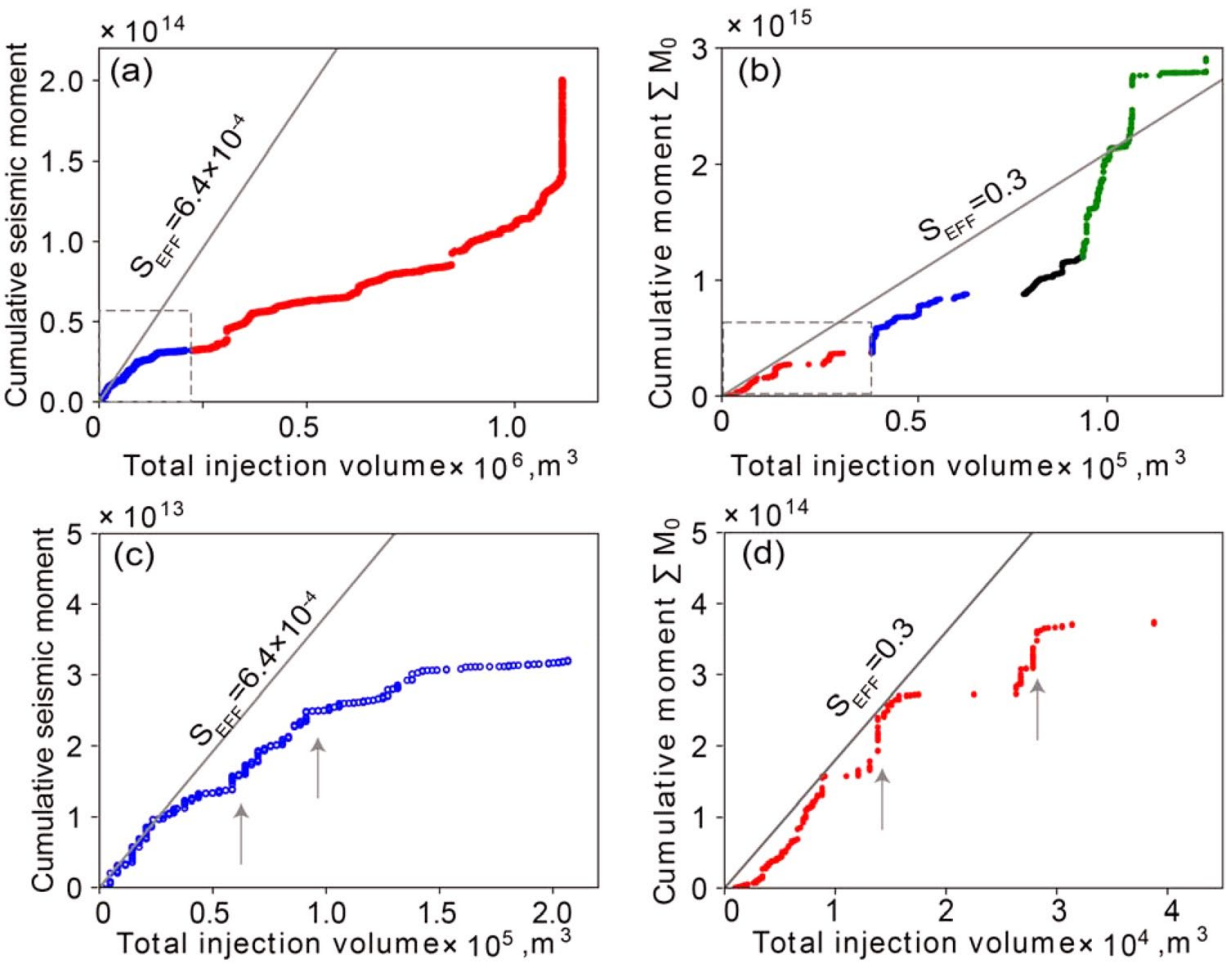
Cumulative seismic moment as a function of total injection volume. (**a**) and (**b**) show the cumulative seismic moment versus total injection volume in project areas 1 and 2, respectively. Slopes to evaluate *S*_EFF_ (grey lines) are determined as the upper bound of the initial sampling data points in project one and project 2, zoomed in (**c**) and (**d**). Arrows indicate changes in slope for the relationship between cumulative seismic moment and total injected volume.

**Figure 5:**
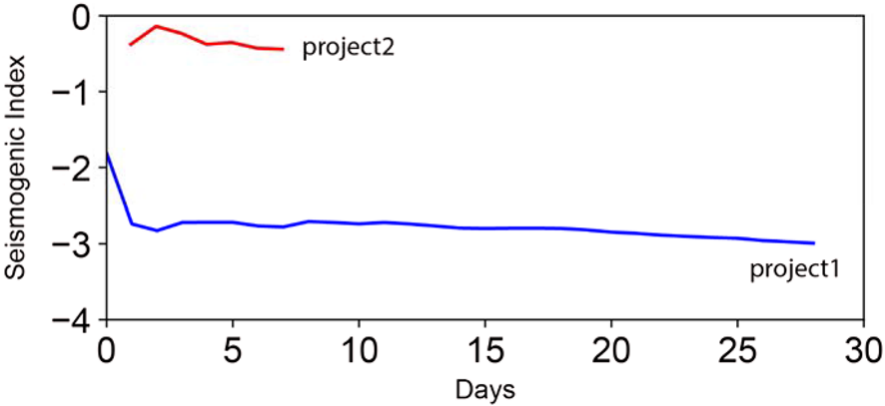
Calculated seismogenic index. Time history of the seismogenic index during sampling periods for project 1 (blue line) and project 2 (red line). The minimum value of Σ during the calibration period is used to forecast the maximum magnitude during the subsequent time period.

Apart from method (1), the methods used here to forecast maximum magnitude require calibration of site-specific parameters (i.e., seismogenic index or *S*_EFF_). To carry out the calibration step, we initially use the initial 20% of the data from the well-injection data and corresponding seismicity data in project area one (highlighted by the grey shaded area in Fig. 2a,b), and data from the first episode in project area two (Fig. 3). To calculate *S*_EFF_ for methods (2) and (4), we use an empirical upper bound for the measured cumulative seismic moment versus injected volume, as illustrated in Fig. 4. To determine the seismogenic index, we computed a time series for Σ and selected the minimum value (Fig. 5). We then tested each of these methods by applying them using the calibrated parameter(s) to hindcast the subsequent seismicity in both project areas to evaluate the associated accuracy for each method. We finally test the robustness of the methods by means of a sensitivity analysis, which uses different episodes from project area two for calibrating the site-specific parameters.

## Results and discussion

Figure 4 shows the cumulative seismic moment as a function of total injection volume for both project areas. The calibration window for each project is highlighted by the dashed box and zoomed in for Fig. 4c,d. Data from both project areas show that the cumulative seismic moment scales non-linearly with total injection volume; in particular, the slope of the cumulative seismic moment versus total injection volume, representing the seismic efficiency ratio (*S*_*EFF*_), usually commences with a relatively large value. After the initial steep slope, the relationship is typically characterized by a repetitive slope change from steep to flat, corresponding to the onset of a period of relative quiescence. Comparison with operational data shows that such step-like changes are often temporally associated with the completion of a stage in the stimulation program. In terms of the underlying physics, induced seismic events are small and frequent at the start of the project, which tends to have a large slope initially. The slope gradually decreases or becomes flat, corresponding to time intervals with relatively few induced earthquakes. Arrows in Fig. 4 point to examples where the slope dramatically increases, indicating a transient higher rate of seismic moment release. As the cumulative seismic moment increases, latency in the system becomes apparent, leading to lag between injection and cumulative moment manifested by flattening of the slope. The latency in the system is due to a time lag between the stimulus and fault response, possibly arising from poroelastic behavior^41^. We apply a line tangent to the upper envelope of the sampling data (grey lines in Fig. 4), implying that subsequent arrested rupture has the potential to release all stored seismic energy and boost the cumulative seismic moment to catch up to the envelope. In this paper, we use a time window that contains 20% of the catalog seismicity events for project one (blue curve in Fig. 4a), and initially episode 1 in project area two (red curve in Fig. 4b) to characterize the initial large *S*_*EFF*_. For consistency with previous studies, methods (1), (2), and (4) were evaluated using *G* = 30 GPa.

**Figure 6:**
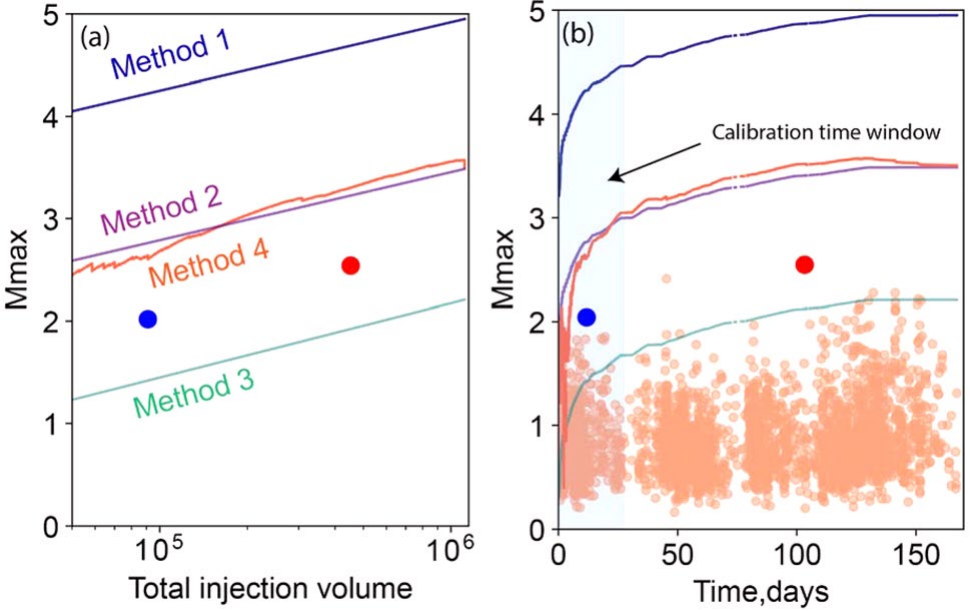
Comparison of estimated upper limits of seismic event magnitude in project area one. (**a**) Estimated *M*_max_ versus total injection volume for the four short-term forecasting methods. Only events above the magnitude of completeness (0.8 *M*_*W*_) are considered for models 2–4. The blue and red symbols show the largest magnitude of induced seismic events during the calibration window and entire dataset, respectively. (**b**) Estimated *M*_max_ versus time. Small orange symbols show observed magnitudes for all events.

**Figure 7:**
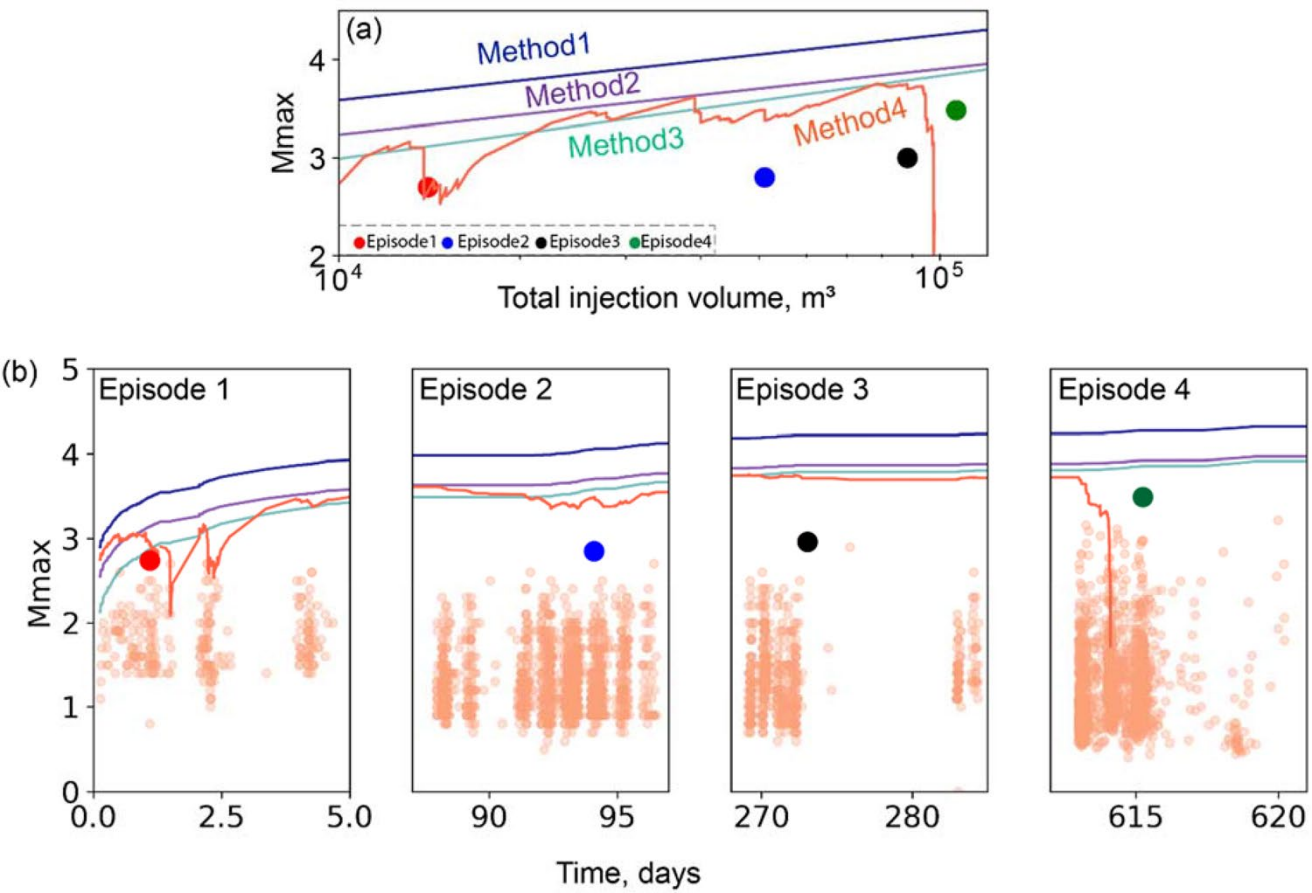
Comparison of estimated upper limits of seismic event magnitude in project area two. (**a**) Estimated *M*_max_ versus total injection volume for the four short-term forecasting methods. Only events above the magnitude of completeness (1.5 *M*_*W*_) are considered for models 1–4. Red, blue, black, and green symbols show the largest observed induced seismic events during each episode. (**b**) Estimated *M*_max_ versus time, zoomed in to each injection episode. Observed magnitudes for all events are shown by small orange symbols, while the large symbol in each panel indicates the largest-magnitude event (by episode).

For project area one, the estimated *M*_max_ for all four methods is plotted versus injected volume in Fig. 6a and versus time in Fig. 6b. In addition, Fig. 6b shows the magnitudes for all observed events, highlighting the largest magnitude during the calibration time window and the entire dataset, respectively. Method one typically overestimates *M*_max_ by approximately two magnitude units, whereas method three typically underestimates *M*_max_ by approximately 0.5 magnitude unit. Methods (2) and (4) forecast similar trends, typically about one magnitude unit greater than the observed maximum magnitude. Similarly, for project area two, the estimated *M*_max_ for all four methods is plotted in Fig. 7. In this case, methods (1) and (3) overestimate *M*_max_, while method (4) predicts the most accurate *M*_max_ for the first three episodes. However, the dense seismic activity and observed magnitudes after episode 3 produces a sharp increase that exceeds the upper limit based on the initial sample dataset of episode 1 (see Table 1). This results in the negative residual seismic moment that invalidates the forecasted *M*_*max*_ during episode 4; as elaborated below, this may be an indicator of a runaway rupture.

**Table 1:** Parameters for sensitivity analysis, calibrated using data from episodes 1–4 of project area two.

	*S*_EFF_	*b*	*M*_*c*_	Σ
Episode 1	0.30	1.18	1.50	− 0.44
Episode 2	0.25	1.27	1.50	− 0.60
Episode 3	0.45	1.36	1.50	− 0.36
Episode 4	3.30	0.95	1.50	− 0.29

In most cases, such as project one and project two in this paper, the upper limit of the cumulative seismic moment due to fluid injection is not fully consumed by induced earthquakes^42^. Consequently, residual elastic energy ($d{M}_{0}$) may be stored in the subsurface, resulting in $d{M}_{0}$ > 0. However, the possibility that the residual seismic moment $d{M}_{0}$ becomes negative cannot be ruled out in cases where (i) significant pre-existing stresses have not been released yet; (ii) the observed earthquakes are not necessarily confined to the region where the crust has been weakened due to fluid injection, as assumed by method (4); and (iii) partitioning between aseismic and seismic deformation may well vary during a stimulation, leading to temporal variation in *S*_EFF_. In the latter case, we remark that a change in *S*_EFF_ sufficient to produce negative $d{M}_{0}$ may be representative of a transition in seismic response and thus constitute a risk indicator. The observed cumulative seismic moment of earthquakes can include events that are induced by poroelastic stress, shear stress change due to mass change^8^, or possibly triggered by (dynamic or static) stress changes or afterslip arising from preceding events^43,44^. This could lead to a larger than predicted cumulative seismic moment, which only considers fluid induced arrested rupture. Similarly, a negative $d{M}_{0}$ could arise when self-arrested ruptures transit abruptly into the runaway ruptures regime^21^ resulting in the release of tectonic strain energy stored in fault systems^45^. As discussed by Rodriguez-Pradilla et al.^46^, the condition ${S}_{EFF}$ > 0.5 indicates runaway rupture—a scenario that is evident during episode 4 (see Table 1 below). In this case, the assumptions for the forecasting methods used here are not valid. Nevertheless, methods (1) and (2) lead to robust estimates for *M*_max_ in the sense that the observed maximum magnitude does not exceed the forecasted value.

### Differences between project areas

Figure 5 shows the calculated time series of the seismogenic index for both projects, based on the cumulative injected volume and the Gutenberg–Richter *b* value, *M*_*c*_, and the cumulative number of seismic events *N* with a magnitude larger than *M*_*c*_. The parameters *b* and *M*_c_ were assumed to be constant, estimated from the calibration windows. There is a fluctuation in Σ at the beginning of both projects, after which it slightly decreases and approaches a constant level. The fluctuation comes from the asynchronous behavior of induced earthquakes and fluid injection (i.e., latency), where seismic events are usually induced after a delay of a few hours to days after injection. It is observed that the (logarithmic) seismogenic index in project area two (− 0.6) is more than two orders of magnitude larger than that in project area one (− 3.0), indicating that for a given injected volume, there is a higher probability for project area two to induce higher-magnitude events.

In project area one, taken as a whole, the fluid is pumped into the reservoir semi-continuously. In contrast, project area two includes four stimulation episodes, each followed by an operational pause ranging from three months to 1 year before the next episode. Our results suggest that such an operational pause does not prevent induced earthquakes. Thus, waiting up to a year may be insufficient for previously elevated pore pressure to fully dissipate for project two due to the low hydraulic diffusion characteristics of the reservoir. Moreover, fluid injection volume (up to approximately 9 × 10^4^ m^3^) from the previous three episodes may increase the shear loading of fractures/faults in episode 4, which leads to a higher probability of inducing higher-magnitude events. This agrees with the largest seismogenic index in episode 4, as shown in Table 1. However, we caution that only two project areas are considered here. Pressure dissipation and fluid withdrawal from hydrocarbon production is expected to reduce the post-stimulation seismogenic potential for months to years. Hence, more observations are required to test these results further.

### Sensitivity analysis

**Figure 8:**
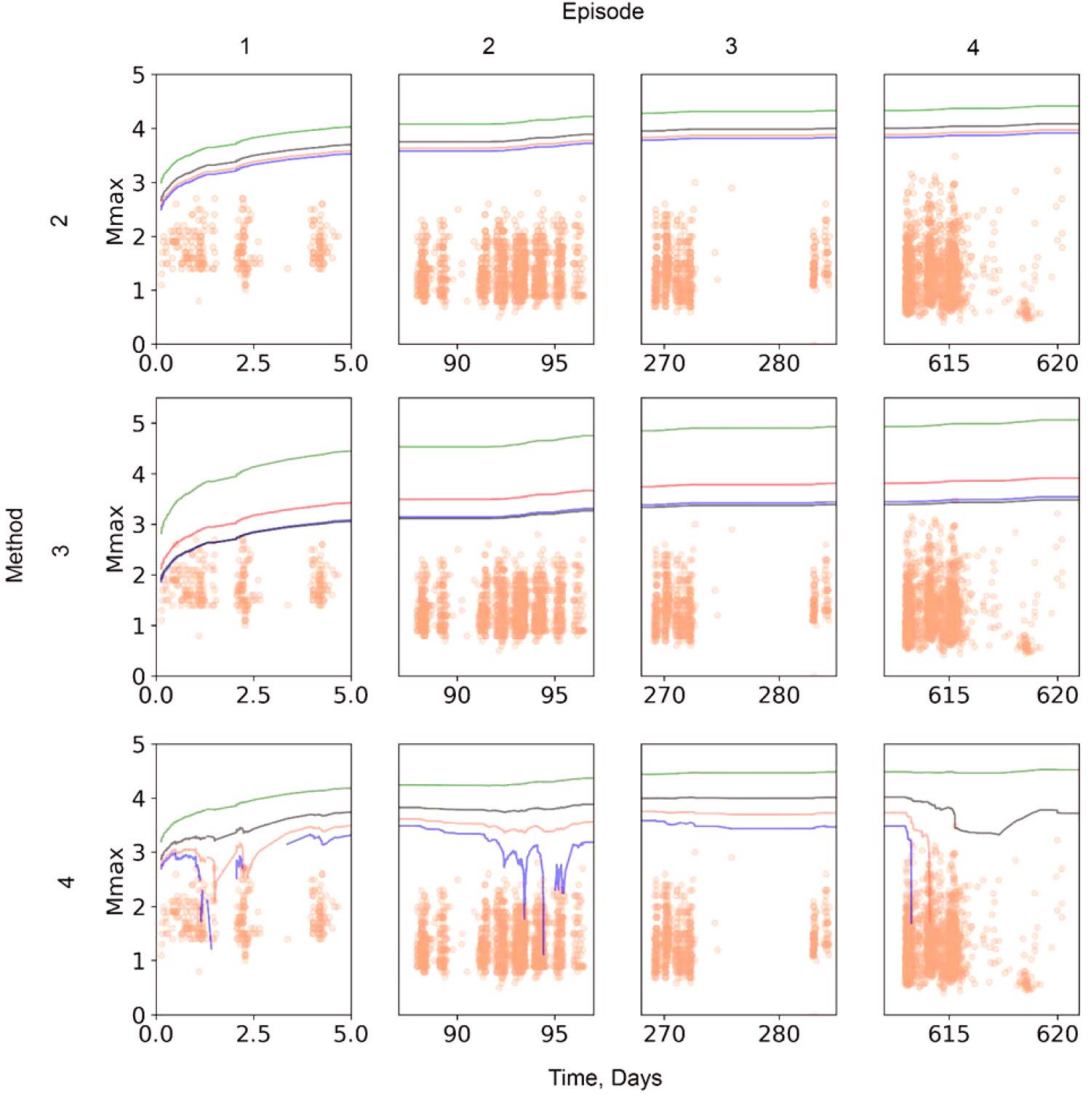
Sensitivity analysis based on the choice of the calibration window. Plots are grouped by method (row) and episode (column). Method one is not considered because it does not require any calibration parameter. Red, blue, black, and solid green lines denote the episode used for calibration, from 1–4, respectively. Orange symbols represent the observed magnitudes.

In project area two, model parameters were evaluated using observations from episode one. The interesting question is, how would the results compare if a different episode were used for calibration of *S*_EFF_? To address this question, Fig. 8 shows a sensitivity analysis in which *M*_max_ estimates are made using calibration parameters derived from different episodes (Table 1) for method (2)–(4). Method (1) is not considered because it does not require any calibration parameter. This analysis assumes that each episode is independent and, hence, not influenced by pore pressure effects nor shear stress changes from other injection episodes.

The sensitivity analysis shows that method (3) is more sensitive to the choice of the calibration window than methods (2) and (4). This is because method (3) requires three calibration parameters: the *b*-value, magnitude of completeness (*Mc*), and seismogenic index (Σ), each with inherent uncertainties, compared with a single calibration parameter (${S}_{EFF}$) for methods (2) and (4). Method (2) predicts a narrower range of *M*_max,_ than method (4). However, it forecasts a maximum magnitude that increases monotonically with the injected volume, which might lead to an overestimation of *M*_max_ especially directly after large seismic events occurred. Figure 8 together with Table 1 shows that the higher ${S}_{EFF}$ or seismogenic index used for calibration, the higher the forecast of *M*_max_ by method (4). This is because our method is dominated by ${S}_{EFF}$, as shown in Eqs. (8) and (9), and despite the fact that the observed cumulative seismic moment is subtracted in real time later. The seismogenic index quantifies the induced seismicity potential, where the higher the seismogenic index, the higher the hazard of induced earthquakes. This is consistent with our predicted limits. We observe that the change in *b* value has no obvious effect on the predicted *M*_max,_ and is not as pronounced as ${S}_{EFF}$ or seismogenic index; consequently, our method and method (2) are relatively insensitive to variations in *b* value.

## Conclusions

In this study, four methods to estimate short-term forecasts of maximum magnitude (*M*_max_) of arrested fault rupture in response to fluid injection during hydraulic fracturing (HF) operations are compared. Method (1) is an often-invoked uncalibrated moment cap, based on various assumptions including that aseismic fault deformation can be neglected. For two case studies that we considered, this method significantly overestimates observed *M*_max_ for hydraulic-fracturing induced seismicity. A second method includes a calibration parameter (*S*_EFF_) that represents the seismic efficiency ratio, i.e., the ratio of the seismic deformation to the total deformation (seismic + aseismic). We calculated this parameter using an initial data sample, by determining an upper-bound value of *S*_EFF_ that provides an envelope to observations during the calibration window. As expected, this approach results in a conservative overestimate of *M*_max_ that is more accurate than the method (1). A third approach, based on sample-size statistics combined with calibration of the seismogenic index (Σ), yields variable results with a high sensitivity to three different calibration parameters. This method underestimates *M*_*max*_ for project area one but overestimates *M*_*max*_ for project area two. Finally, we tested a new method that forecasts *M*_max_ for arrested rupture based on the residual seismic moment, based on a calibrated cumulative seismic moment cap approach. Unlike methods (1)–(3), which forecast a maximum magnitude that increases monotonically with the injected volume, our new method (4) differs from previous approaches by continuously tracking the temporal changes in cumulative seismic moment. It refines method (2) to achieve a tighter upper bound on the seismic hazard. Our results show that in most cases, method (4) provides the most accurate short-term expected *M*_max_ (4 out of 6 forecast windows). However, it is also sensitive to the choice of calibration window and can lead to under-prediction of *M*_max_ in cases where the residual seismic moment becomes negative. Since it invalidates the underlying assumption of arrested rupture, we propose that the occurrence of negative $d{M}_{0}$ arising during the application of method (4) is of practical utility as it may signal a departure from stable, arrested events and the onset of a runaway rupture. Establishing this systematically remains a challenge for the future.

## Data Availability

Codes supporting the findings of this manuscript are available from the corresponding author. Data from the injections and seismicity cannot be publicly released but are visualized in the corresponding figures and tables, including the supporting information.
